# Connecting Gene Variation to Treatment Outcomes in Metastatic Castration-Resistant Prostate Adenocarcinoma: Insights into Second-Generation Androgen Receptor Axis-Targeted Therapies

**DOI:** 10.3390/ijms25189874

**Published:** 2024-09-12

**Authors:** Ana Vaz-Ferreira, Valéria Tavares, Inês Guerra de Melo, Patrícia Rafaela Rodrigues, Ana Afonso, Maria Joaquina Maurício, Rui Medeiros

**Affiliations:** 1 Oncology Department, Portuguese Institute of Oncology of Porto (IPO Porto), 4200-072 Porto, Portugal; ana.vaz.f@gmail.com (A.V.-F.); patricia.rafaela.rodrigues@ipoporto.min-saude.pt (P.R.R.); ana.freitas.afonso@ipoporto.min-saude.pt (A.A.); jmauricio@ipoporto.min-saude.pt (M.J.M.); 2 Molecular Oncology and Viral Pathology Group, Research Center of IPO Porto (CI-IPOP)/Pathology and Laboratory Medicine Department, Clinical Pathology SV/RISE@CI-IPOP (Health Research Network), Portuguese Oncology Institute of Porto (IPO Porto)/Porto Comprehensive Cancer Centre (Porto CCC), 4200-072 Porto, Portugal; valeria.tavares@ipoporto.min-saude.pt (V.T.); ines.melo@ipoporto.min-saude.pt (I.G.d.M.); 3 Faculty of Medicine of University of Porto (FMUP), 4200-072 Porto, Portugal; 4 ICBAS—Instituto de Ciências Biomédicas Abel Salazar, Universidade do Porto, 4050-313 Porto, Portugal; 5 Faculty of Health Sciences, Fernando Pessoa University, 4200-150 Porto, Portugal; 6 Research Department, Portuguese League Against Cancer (NRNorte), 4200-172 Porto, Portugal

**Keywords:** prostatic neoplasms, castration resistance, abiraterone acetate, enzalutamide, treatment outcomes, SNPs

## Abstract

Prostate cancer (PC) is one of the most commonly diagnosed tumours among men. Second-generation androgen receptor axis-targeted (ARAT) agents, namely abiraterone acetate (AbA) and enzalutamide (ENZ), are currently used in the management of metastatic castration-resistant PC (mCRPC). However, the treatment is challenging due to the lack of prognostic biomarkers. Meanwhile, single-nucleotide polymorphisms (SNPs) have emerged as potential prognostic indicators of mCRPC. Thus, this study evaluated the impact of relevant SNPs on the treatment outcomes of 123 mCRPC patients enrolled in a hospital-based cohort study. The *CYP17A1* rs2486758 C allele was associated with a 50% reduction in the risk of developing castration resistance (hazard ratio (HR) = 0.55; *p* = 0.003). Among patients without metastasis at tumour diagnosis and under AbA, a marginal association between *YBX1* rs10493112 and progression-free survival was detected (log-rank test, *p* = 0.056). In the same subgroup, significant associations of *HSD3B1* rs1047303 (CC/CA vs. AA; HR = 3.41; *p* = 0.025), *YBX1* rs12030724 (AT vs. AA; HR = 3.54; *p* = 0.039) and *YBX1* rs10493112 (log-rank test, *p* = 0.041; CC vs. AA/AC; HR = 3.22; *p* = 0.053) with overall survival were also observed, which were confirmed by multivariate Cox analyses. Although validation with larger cohorts is required, these findings suggest that SNPs could enhance the prognosis assessment of mCRPC patients, leading to a more personalised treatment.

## 1. Introduction

In 2020, prostate cancer (PC) stood as the second leading malignancy and the fifth cancer-related cause of death among men worldwide, with approximately 1.4 million new cases and 400,000 deaths [1]. In Portugal, according to the national oncology registry, PC was the most frequent cancer in men in the same year, with 5776 new cases and a mortality rate of 39.3 deaths per 100,000 men [2]. Given its high incidence and mortality, this malignant disease represents an important public health problem [3].

Men with metastatic PC at diagnosis or those who have relapsed after a radical therapeutical approach are usually treated with salvage radiation, chemotherapy, and androgen deprivation therapy (ADT) [4]. The latter represents the standard treatment for advanced PC. This therapeutical approach involves medically or surgically blocking androgen generation or directly inhibiting androgen receptors (ARs) to induce castration, defined as serum levels of testosterone ≤50 mg/dL [5,6]. Most PC patients initially respond to castration; however, disease progression is practically inevitable [7,8]. Although not fully understood, the de novo synthesis of androgens and AR overexpression by tumour cells, as well as the activation of signalling pathways that mediate cell proliferation, may enhance the acquisition of castration resistance [8]. 

In recent years, second-generation androgen receptor axis-targeted (ARAT) agents have been developed, including abiraterone acetate (AbA) and enzalutamide (ENZ). Although advanced PC is still incurable, these therapeutical agents have shown a significant benefit to patients’ overall survival (OS) when used in the treatment of metastatic castration-resistant prostate cancer (mCRPC) [4]. While both agents target the androgen signalling pathway, their mechanisms of action differ. AbA is a selective inhibitor of androgen biosynthesis through the potential and irreversible blockade of cytochrome P450 family 17 subfamily A member 1 (CYP17A1), resulting in the absence of androgens in the blood and within tumour cells [8,9]. On the other hand, ENZ is an antiandrogen that competitively blocks the binding of androgens to their receptor and inhibits the translocation of the activated receptor–ligand complex to the nucleus [10,11]. The choice between AbA and ENZ is mainly grounded on their toxicity profile, patients’ medical background and concomitant medications [12].

Genetic polymorphisms refer to variations in the DNA sequence that occur at a frequency of 1% or more in a given population. These variations can impact the susceptibility to common and complex disorders, disease severity and even the response to established therapies [13]. Understanding how genetic polymorphisms influence therapeutic response is an active research field, as it might enable more personalised and effective treatment strategies [14,15]. Inclusively, several genetic polymorphisms have been investigated in the context of PC, including variations in genes related to ARs, drug metabolism, DNA repair pathways and cellular signalling pathways. As anticipated, those in genes related to the androgen signalling pathway are reported to affect the response to androgen-targeted therapies [16,17]. Numerous single-nucleotide polymorphisms (SNPs) have been studied in the context of response to treatment in the hormone-sensitive phase and castration resistance in PC patients treated with ARAT agents [13]. However, the existing data are inconsistent and external validation is required. From an oncologist’s perspective, dissecting the influence of these markers on the response to hormonal treatment could aid in the identification of patients at high risk for the development of castration resistance and therapeutic failure regarding ARAT agents, allowing a more individualised clinical decision-making. To date, the most widely used biomarker to monitor PC progression and treatment response is prostate-specific antigen (PSA), a serine protease expressed in the prostatic epithelium that is upregulated in PC. However, benign conditions, such as prostate hypertrophy and prostate infection, may also lead to high serum levels of PSA, affecting its specificity [18,19]. Hence, while PSA remains crucial, integrating additional biomarkers, including clinical and biological factors, is necessary to enhance the accuracy of prognostic models. Thus, given the implications of SNPs in PC pathways, this study investigated the influence of these genetic determinants on treatment outcomes of mCRPC patients from the North Region of Portugal (Iberian Peninsula, Southwestern Europe).

## 2. Results

### 2.1. Population Characteristics

A total of 123 mCRPC patients were enrolled in this retrospective hospital-based cohort study (Table 1). The median follow-up time was 28 months (minimum = 3 months; maximum = 76 months). The patients had a mean age of 66.3 ± 8.4 years upon initiating ARAT-based treatment and most of them did not present metastatic disease at tumour diagnosis (N = 86; 69.9%). The median time from tumour diagnosis to the development of castration resistance was 6.7 years (minimum = 0.7 years; maximum = 27 years). In the cohort, 40 patients were treated with AbA and the remaining 83 with ENZ for mCRPC management. There were no significant differences in patients’ characteristics according to ARAT agent (χ^2^, *p* > 0.05; Table 1). Likewise, no significant differences in progression-free survival (PFS) (PSA or radiological) or OS were found between the two treatment groups (log-rank test, *p* > 0.05).

### 2.2. SNPs and Their Genotype Distribution

No significant differences in the genotype distribution of the evaluated SNPs according to the ARAT agent were detected (χ^2^, *p* > 0.05; Table 2). Considering the entire cohort of mCRPC patients (N = 123), the distribution of the genotypes was comparable to that described in the European population and the Iberian subpopulation.

### 2.3. SNPs and Treatment Outcomes

**Figure 1:**
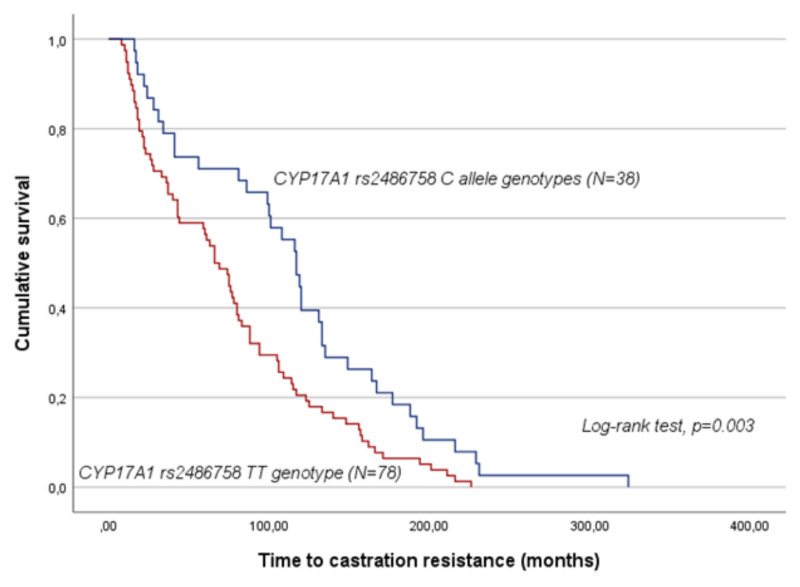
Time to castration resistance (TCR) by Kaplan–Meier and Log-rank test for mCRPC patients (N = 116), according to *CYP17A1* rs2486758 genotypes. Patients with the C allele had a prolonged TCR compared to those carrying the TT genotype (*p* = 0.003).

Considering the entire cohort, *CYP17A1* rs2486758 was significantly associated with the time to castration resistance (TCR) under a dominant genetic model. Patients carrying the C allele (minor allele) exhibited a prolonged TCR compared to their counterparts (CT/CC vs. TT; mean TCR of 115.2 ± 11.7 months and 76.3± 6.5 months, respectively; log-rank test, *p* = 0.003; Figure 1). According to univariate Cox analysis, the C allele is associated with a 50% reduction in the risk of developing castration resistance (hazard ratio (HR) = 0.55; 95% confidence interval (CI), 0.37–0.82; *p* = 0.003). As for the remaining SNPs, no significant association was observed even when stratifying the analysis based on the tumour extension at diagnosis (log-rank test, *p* > 0.05).

Concerning PFS, no significant impact of the evaluated SNPs was found in the overall cohort (log-rank test, *p* > 0.05). The same was observed in stratified analyses according to the ARAT agent. However, focusing on patients with localised tumour at diagnosis, a marginal association between the polymorphism *Y-box binding protein 1* (*YBX1*) rs10493112 and PFS was detected among those treated with AbA. Specifically, patients carrying the AA genotype presented a lower time to disease progression than C allele carriers (AA vs. CC/AC; mean PFS of 8.3 ± 4.7 months and 17.8 ± 2.8 months, respectively; log-rank test, *p* = 0.056).

**Figure 2:**
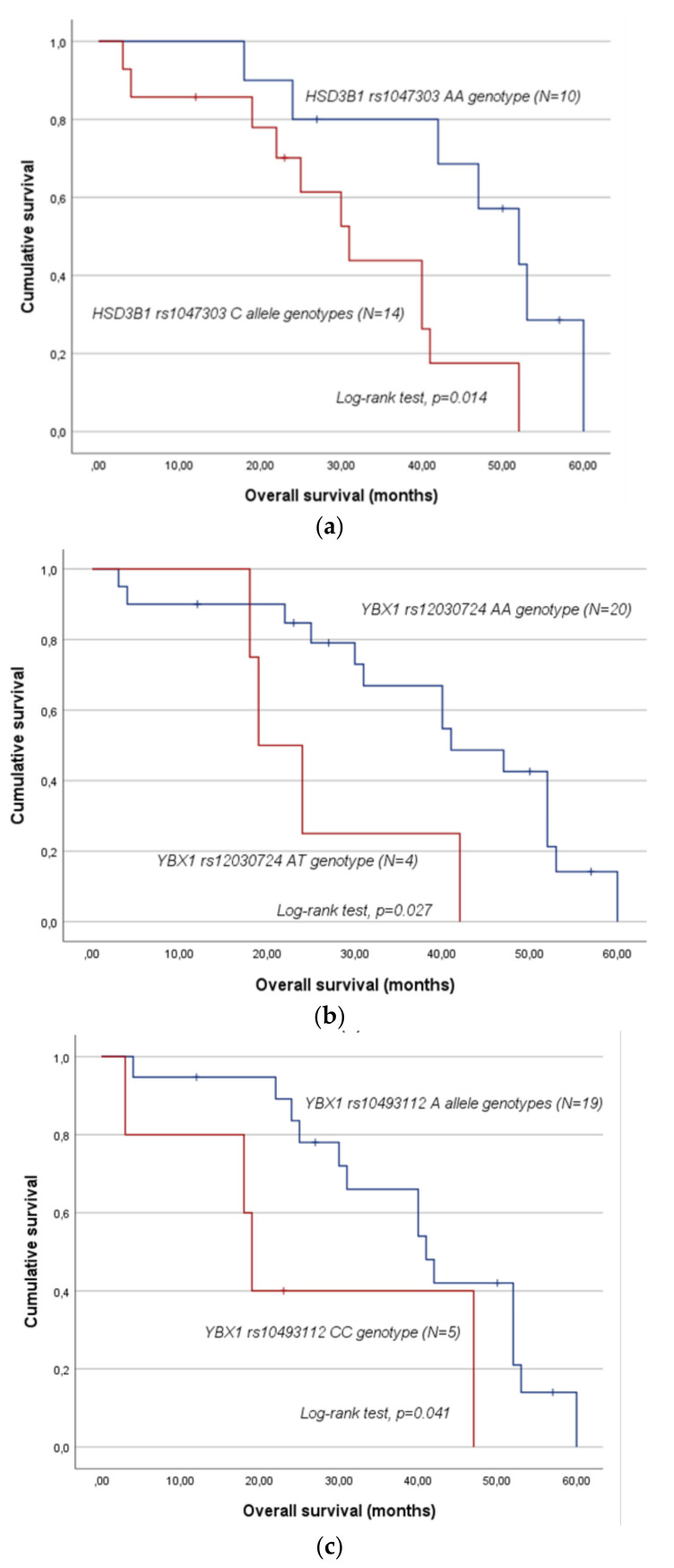
Overall survival (OS) by Kaplan–Meier and Log-rank test for mCRPC patients with localised tumour at diagnosis and under AbA-based treatment (N = 24), according to *HSD3B1* rs1047303 (**a**), *YBX1* rs12030724 (**b**) and *YBX1* rs10493112 (**c**) genotypes. Patients with the *HSD3B1* rs1047303 C allele genotypes had a lower OS compared to those carrying the AA genotype (*p* = 0.014). Patients with the *YBX1* rs12030724 AT genotype had a lower OS than AA genotype carriers (*p* = 0.027). Patients with the *YBX1* rs10493112 CC genotype had a lower OS than their counterparts (*p* = 0.041).

Regarding OS, no association with the evaluated SNPs was detected either in the entire cohort or within the subgroups according to the ARAT agent used (log-rank test, *p* > 0.05). Nevertheless, among patients with localised disease at cancer diagnosis and treated with AbA for mCRPC management, significant associations were found concerning *hydroxy-delta-5-steroid dehydrogenase, 3 beta-and steroid delta-isomerase 1 (HSD3B1)* rs1047303 (CC/CA vs. AA; log-rank test, *p* = 0.014; Figure 2a), *YBX1* rs12030724 (AT vs. AA; log-rank test, *p* = 0.027; Figure 2b) and *YBX1* rs10493112 (CC vs. AA/AC; log-rank test, *p* = 0.041; Figure 2c). Carriers of the *HSD3B1* rs1047303 C allele genotypes presented a lower survival compared with those with the AA genotype (mean OS of 31.0 ± 4.4 months and 46.5 ± 4.9 months, respectively), which was confirmed by univariate Cox analysis (CC/CA vs. AA; HR = 3.41; 95% CI 1.17–9.93; *p* = 0.025). The same was observed for the *YBX1* rs12030724 AT genotype. Those with this genotype had a lower OS than AA carriers (mean OS of 25.8 ± 5.6 months and 40.2 ± 4.0 months, respectively). This result was also corroborated by univariate Cox analysis (AT vs. AA; HR = 3.54; 95% CI 1.07–11.71; *p* = 0.039). The *YBX1* rs10493112 CC genotype also demonstrated a negative impact on patients’ OS compared to the A allele genotypes (mean OS of 26.8 ± 9.0 months and 40.6 ± 3.7 months, respectively). In the univariate Cox analysis, a marginal association was detected (CC vs. AA/AC; HR = 3.22; 95% CI 0.98–10.51; *p* = 0.053). Multivariate Cox analyses adjusted for Gleason score (>7 vs. ≤7) and PSA levels (≥18 vs. <18 ng/mL) at tumour diagnosis corroborated the impact of these three SNPs on patients’ OS (Table 3).

## 3. Discussion

ADT is the cornerstone of metastatic PC; however, castration resistance is inevitable, imposing a detrimental impact on patients’ survival [20]. Patients with mCRPC are standardly treated with docetaxel-based chemotherapy or second-generation ARAT agents, like AbA and ENZ. Multiple phase 3 trials have demonstrated that these agents improve both PFS and OS for mCRPC patients treated with these agents, whether as a first choice or following docetaxel-based chemotherapy [21]. The treatment efficacy with AbA and ENZ is thought to be influenced by genetic variants [22]. While some associations have been pinpointed, no genetic markers have yet been incorporated into clinical practice to guide the therapeutical decision-making [13]. To provide more insights into the topic, the present study was designed to investigate the impact of relevant SNPs on the treatment outcomes of mCRPC patients treated with AbA or ENZ.

No significant differences in PFS (PSA or radiological) and OS according to the ARAT agent used were observed, which is supported by the existing literature [21]. Regarding the SNPs, starting with rs2486758 (c.-362T > C), this is a regulatory region SNP that consists in the substitution of a thymine (T) for a cytosine (C) in *CYP17A1* (chromosome 10q24.32) [23]. This gene encodes for a steroidogenic enzyme with the same name, which is a member of the cytochrome p450 family expressed in testicular Leydig cells and the adrenal cortex [24]. More than 50% of human PCs express CYP17A1 [25]. This enzyme has a key role in PC progression, allowing the tumour cells to synthesise androgens intracellularly, switching from a hormone-sensitive to a hormone-resistant phenotype, thus bypassing ADT [26]. Given its location and the existing data, the SNP rs2486758 is thought to modulate the transcriptional activity of *CYP17A1*, interfering with the enzyme levels [14,27,28]. In this study, patients carrying the C allele exhibited a lower TCR than those with the TT genotype. This finding corroborates that CYP17A1 is relevant for the acquisition of castration resistance. As for patients’ PFS and OS, no association was found regardless of disease extent at PC diagnosis and the ARAT agent used. Interestingly, in a retrospective study conducted by Crucitta et al. (2020) with mCRPC patients under AbA, the SNP C allele was associated with a shorter PFS compared with its counterpart [29]. This finding was later confirmed by Ferrero et al. (2023) [14]. Previously, Iverson et al. (2012) showed that healthy premenopausal women with the SNP C allele exhibited higher levels of 17β-oestradiol levels [30]. As CYP17A1 is implicated in the synthesis of both estrogenic and androgenic steroids, men carrying the rs2486758 C allele may present higher intra-tumoural testosterone levels compared to their counterparts, which could explain the findings of Crucitta et al. (2020) and Ferrero et al. (2023) [14]. Hence, considering the results of the present study and the existing literature, there is a seemly conflicting impact of the C allele in TCR and PFS, which should be further explored in larger cohorts to dissect the implications of rs2486758 among mCRPC patients under ARAT agents.

The polymorphism rs1047303 (1245 A > C) is a missense SNP that consists of the substitution of an adenine (A) for a cytosine (C) in *HSD3B1* (chromosome 1p12) [23]. This gene encodes for 3β-hydroxysteroid dehydrogenase-1 (3βHSD1), an essential enzyme involved in dihydrotestosterone (DHT) synthesis in adrenal tissue in PC [31,32]. Importantly, 3βHSD1 converts AbA in multiple downstream metabolites, including Δ4-abiraterone (D4A), which strongly inhibits steroidogenic enzymes, such as CYP17A1 and even AR itself. Nevertheless, metabolites of D4A have a pro-androgenic activity, which might explain the limitations of AbA efficacy [31]. The SNP rs1047303 has been associated with functional differences in 3βHSD1, influencing androgen production. Specifically, two functional forms of the enzyme are recognised: an adrenal-permissive (rs1047303 C allele) and an adrenal-restrictive (rs1047303 A allele). While the former allows for a rapid DHT synthesis, the latter limits androgen production [31]. However, few studies have been conducted to investigate the impact of the SNP on the clinical outcomes of mCRPC patients treated with ARAT agents. A Japanese study suggested that men who carry the variant allele (C allele) tend to become resistant to ADT but without significant differences in OS [32]. In addition, the same study showed that the combined variation in *HSD3B1* and *steroid 5 alpha-reductase 2* (*SRD5A2*) genes augmented the ability of prognostic stratification and may be a predictive factor for treatment efficacy with AbA due to their specificity to encode enzymes for AbA metabolism, but not for other therapeutical agents, such as ENZ and docetaxel [32]. Furthermore, results from two prospective studies with a total of 547 mCRPC patients treated with AbA or ENZ show that the *HSD3B1* rs1047303 CC genotype is associated with potentially worse outcomes, including lower time to disease progression and smaller PSA response rates, independently of the treatment chosen [33]. In this present study, among mCRPC patients with non-metastatic disease at diagnosis and under AbA-based treatment, a significant association between the rs1047303 variant and patients’ OS was detected (log-rank test, *p* = 0.014). Namely, those carrying the C allele genotypes present a lower survival than their counterparts. This finding, which is consistent with the literature, was corroborated by both univariate (CC/CA vs. AA; HR = 3.41; *p* = 0.025) and multivariate (CC/CA vs. AA; adjusted HR (aHR) = 3.28; *p* = 0.037) Cox analyses. As for TCR and PFS, no significant association was detected.

The SNP rs12030724 is an intronic variant characterised by the substitution of an adenine (A) for a thymine (T) in *YBX1* (chromosome 1p34.2) [23]. This gene encodes for Y-box binding protein-1 (YB-1), a multifunctional protein that acts in the nucleus as a transcription factor and can regulate gene expression in the cytoplasm. Several studies have shown that YB-1 is overexpressed in various neoplasms and interferes with treatment resistance mechanisms [34]. In the setting of mCRPC, YB-1 expression is upregulated, driving castration resistance by regulating ARs and their sensitivity to pharmaceutical agents [35]. In a Japanese population analysed by Shiota et al. (2021), men with the rs12030724 AT/TT genotypes presented a lower progression risk, as well as a lower risk of any-cause mortality when compared with AA genotype carriers [36]. In the present study, considering the patients with non-metastatic disease at diagnosis and treated with AbA, those with the AT genotype had a lower OS than their counterparts, a finding that was corroborated by univariate (AT vs. AA; HR = 3.54; *p* = 0.039) and multivariate (AT vs. AA; aHR = 4.43; *p* = 0.025) Cox analyses. Thus, this result is not in line with Shiota et al. (2021). Furthermore, no significant association was detected between *YBX1* rs12030724 and patients’ TCR and PFS, respectively. Given the limited body of evidence, more studies will be required to dissect the biological plausibility of this finding. 

Like rs12030724, the SNP rs10493112 is also an intronic variant that lies within *YBX1*. This variant consists of the substitution of a cytosine (C) for an adenine (A) [23]. In a previous study performed by our research group, this *YBX1* polymorphism was shown to influence PFS in mCRPC patients treated with AbA [37]. Specifically, AA genotype carriers showed a higher risk of disease progression than the CC/CA patients (Cox regression analysis, *p* = 0.009). In the present study, a marginally significant association between the variant rs10493112 and patients’ PFS was found, focusing on those with non-metastatic disease at PC diagnosis and under treatment with AbA (log-rank test, *p* = 0.056). Namely, the patients with the rs10493112 AA genotype tend to progress faster, which corroborates our previous results. In the same subgroup, a significant impact on patients’ OS was also detected (log-rank test, *p* = 0.041). Specifically, the rs10493112 CC genotype demonstrated a negative impact on patients’ survival compared to the A allele genotypes, a finding that was confirmed by both univariate (CC vs. AA/AC; HR = 3.22; *p* = 0.053) and multivariate (CC vs. AA/AC; aHR = 5.50; *p* = 0.022) Cox analyses. As previously mentioned, YB-1 is regarded as a multifunctional protein, potentially influencing several biological pathways implicated in both physiological and pathological settings. Although this might help explain the distinct impact of rs12030724 genotypes on mCRPC patients’ PFS and OS, future studies in larger cohorts are required to better comprehend the implications of this genetic variant. As for TCR, no significant association concerning this SNP was observed. 

Overall, the study’s findings indicate that SNPs could impact the clinical outcomes of mCRPC patients receiving ARAT agents, especially AbA. In the context of advancing liquid biopsy technologies, integrating these genetic factors with key clinical parameters, such as PSA levels and Gleason score, may lead to the development of more personalised treatment approaches, thereby improving the precision of mCRPC management. However, this study had some limitations that need to be acknowledged. First, the cohort size was small, which may have limited the statistical power. Furthermore, the retrospective nature of the study led to missing information. The study’s limited follow-up duration may also have hindered a comprehensive understanding of the prognostic value of assessed SNPs over time due to the long-term nature of mCRPC management. Lastly, potential confounding variables affecting patient prognosis, including lifestyle, environmental exposures, and genetic variations, could have been overlooked.

## 4. Materials and Methods

### 4.1. Study Design and Population Description

A retrospective, single-centre, hospital-based cohort study was conducted enrolling mCRPC patients treated at the Portuguese Institute of Oncology of Porto (IPO Porto). The patients were recruited between May 2022 and January 2023 if they had a confirmed histopathological diagnosis of PC, were 18 years of age or older, and were currently or previously treated with AbA or ENZ for mCRPC management. Those with other active tumours were excluded. 

Medical records of the enrolled patients were consulted to collect their demographic and clinical characteristics, as well as the follow-up data. Notably, during the study period, serum levels of total PSA were consistently measured at our institute using the Elecsys chemiluminescence immunoassay (Roche, Mannheim, Germany) on the Roche Diagnostics Cobas e 801 immunoassay analyser.

Informed written consent was obtained from each patient. The study was conducted according to the principles of the Helsinki Declaration and approved by the ethics committee of IPO Porto (CES. 198/018, 12 July 2018).

### 4.2. Sample Collection and DNA Extraction

Peripheral venous blood samples from each patient were collected in EDTA-containing tubes (BD Vacutainer Blood Collection Tube, Becton Dickinson, NJ, USA). Genomic DNA was extracted from nucleated cells using the QIAamp DNA Blood Mini Kit (Cat. No. 51106, Qiagen, Hilden, Germany), following the manufacturer’s instructions.

### 4.3. SNP Selection and Genotyping

A literature review of SNPs associated with castration resistance among PC patients and/or response to AbA and ENZ was conducted. Considering a minor allele frequency (MAF) of at least 10%, four SNPs were selected to be evaluated, including *CYP17A1* rs2486758, *HSD3B1* rs1047303 and two variants from the *YBX1*: rs12030724 and rs10493112. 

SNP genotyping was conducted in a StepOne Plus Real-time PCR system (Applied Biosystems®, Foster City, CA, USA). Employing the TaqMan^®^ Allelic Discrimination method, each PCR reaction was performed using 2.5 µL of TaqPath^TM^ ProAmp^TM^ Master Mix (1x), 2.375 µL of sterile water, 0.125 µL of TaqMan™ Genotyping Master Mix (C__15807798_10 for *CYP17A1* rs2486758, C__8695674_10 for *HSD3B1* rs1047303, C_30860318_20 for *YBX1* rs12030724 and C_30509500_10 for *YBX1* rs10493112) and 1.0 µL of genomic DNA, in a total volume of 6 µL. The thermal cycling conditions for DNA amplification were previously described [37]. Negative controls (without DNA) were incorporated into each PCR reaction to prevent false positives. A double sampling for at least 20% of randomly selected DNA samples was also conducted, with an accuracy of over 99%. Data on DNA amplification were analysed using the StepOne Software (version 2.3 Applied Biosystems®, Foster City, CA, USA). Two independent researchers evaluated the results without access to the demographic and clinicopathological data of the patients enrolled in the study.

### 4.4. Statistical Analysis

Data analysis was conducted using IBM SPSS Statistics for Windows version 29 (IBM Corp., 2020, Armonk, NY, USA).

The chi-square test (χ^2^) was employed to assess the associations between categorical variables. For continuous variables, depending on the variable distribution assessed by the Kolmogorov–Smirnov test (N > 50), either the Student’s *t*-test or the Mann–Whitney U test was applied to compare the differences (Table 1).

The PSA response was defined as a decrease of ≥50% in the PSA concentration from the pre-treatment baseline value, confirmed after ≥4 weeks by an additional PSA evaluation. PSA progression was defined as after a decline from baseline, an increase that was ≥25% and ≥2 ng/mL above the nadir, confirmed by a second value ≥3 weeks later or, if no decline from baseline was achieved, an increase that was ≥25% and ≥2 ng/mL from baseline beyond 12 weeks. Radiological response or progression was defined according to modified Response Evaluation Criteria in Solid Tumours (RECIST) criteria version 1.1. [38] or based on bone scans according to criteria adapted from the PCWG3 [6].

Three measures of clinical outcome were considered, namely TCR, PFS and OS. The former was defined as the interval between PC diagnosis and acquisition of castration resistance. PFS was deemed the time from initiation of treatment with AbA or ENZ to the occurrence of any type of progression (radiographic or PSA) or death, whichever happened first. On the other hand, OS was defined as the time between the date of ARAT treatment initiation and the date of death due to any cause. Patients showing no event (death or progression) or lost to follow-up were censored at the date they were last known to be alive. 

The influence of the evaluated SNPs on the clinical outcomes was assessed using the Kaplan–Meier method and Cox regression analysis. After an initial assessment of the survival curves of each SNP under the additive genetic model, the most suitable model was selected for further analyses. Stratified analyses based on tumour extension at diagnosis and the ARAT agent used were conducted. For significant associations according to Cox univariate analyses, multivariate analyses were conducted adjusting for Gleason (>7 vs. ≤7) and PSA levels (≥18 vs. <18 ng/mL) at disease diagnosis.

In all analyses, a *p*-value lower than 0.05 was considered statistically significant.

## 5. Conclusions

Research on the influence of genetic polymorphisms on the prognosis of mCRPC patients holds promise for advancing precision medicine in disease management, leading to more tailored and effective therapies to improve outcomes for these patients. However, this research field faces many challenges. These include the need for larger, well-characterised patient cohorts, standardised genetic testing methods, validation of identified genetic markers across different populations, and integration of genetic data into clinical decision-making processes. In this study, the impact of four relevant SNPs on mCRPC patients’ prognosis was assessed. The variant *CYP17A1* rs2486758 was implicated in the risk of developing castration resistance. *HSD3B1* rs1047303 and *YBX1* rs12030724 significantly impacted patients’ OS among those with non-metastatic disease at diagnosis and under AbA-based treatment for mCRPC. In the same subgroup, *YBX1* rs10493112 was shown to impact PFS and OS. Altogether, these four genetic variants seem to be valuable prognostic biomarkers for mCRPC patients with potential clinical applicability. Despite the interesting results, external validation with larger cohorts is required, particularly given the limited body of evidence. The investigation should also be extended to include other genetic variants that could be relevant in PC pathways. Consideration should also be given to the inclusion of other more recently approved ARAT agents, including Apalutamide and Darolutamide, in future studies, as these treatments hold the potential for broadening the scope and applicability of the findings [39].

## Figures and Tables

**Table 1:** Description of the mCRPC patients enrolled in the study according to AbA or ENZ treatment (N = 123).

	AbA (N = 40)	ENZ (N = 83)	*p*-Value
Age at ARAT agent initiation (years) *	66.6± 8.7	66.1 ± 8.4	0.794
**Gleason score at PC diagnosis (N, %)**			
≤7	18 (45.0)	39 (47.0)	0.989
>7	22 (55.0)	44 (53.0)	
**Extent of disease at PC diagnosis (N, %)**			
Localised	24 (61.5)	62 (77.5)	0.108
Metastatic	15 (38.5)	18 (22.5)	
PSA at PC diagnosis (ng/mL) **	18.4 [4.9–3183.0]	18.0 [1.4–8039.0]	0.826
**Primary cancer therapy (N, %)**			
Surgery	14 (35.0)	32 (39.0)	0.817
Radiotherapy alone	4 (10.0)	7 (8.5)	1.000
Radiotherapy + Hormonal treatment	4 (10.0)	12 (14.6)	0.670
Hormonal/orchidectomy	15 (37.5)	23 (28.0)	0.395
Hormonal treatment + Chemotherapy	3 (7.5)	8 (9.8)	0.943
**Extent of disease at ARAT start (N, %)**			
Bone only	33 (82.5)	67 (80.7)	0.780
Soft tissue or node	22 (55.0)	50 (60.2)	0.721
Visceral	1 (2.5)	0	0.708
Other	2 (5.0)	1 (1.2)	0.513
**ECOG PS baseline**			
0–1	36 (90.0)	79 (95.2)	0.483
2	4 (10.0)	4 (4.8)	
PSA baseline (ng/mL) **	32.1 [1.7–597.0]	28.6 [0.02–2143.0]	0.847
**Radiological response**			
Complete response	5 (12.8)	3 (4.0)	0.820
Partial response	1 (2.6)	11 (14.7)	
Stable disease	27 (69.2)	53 (70.7)	
Progressive disease	5 (12.8)	4 (5.3)	
Omitted	1 (2.6)	4 (5.3)	
**PSA response (N, %)**	33 (82.5)	70 (93.3)	0.136
***CYP17A1* rs2486758 polymorphism (N, %)**			
TT	25 (62.5)	59 (71.1)	0.430
TC	13 (32.5)	20 (24.1)	
CC	2 (5.0)	4 (4.8)	
***HSD3B1* rs1047303 polymorphism (N, %)**			
AA	17 (42.5)	42 (50.6)	0.540
AC	21 (52.5)	36 (43.4)	
CC	2 (5.0)	5 (6.0)	
***YBX1* rs12030724 polymorphism (N, %)**			
AA	33 (82.5)	71 (85.5)	0.662
AT	7 (17.5)	12 (14.5)	
TT	-	-	
***YBX1* rs10493112 polymorphism (N, %)**			
AA	9 (22.5)	16 (19.3)	0.188
AC	23 (57.5)	37 (44.6)	
CC	8 (20.0)	30 (36.1)	

The table includes data regarding initial tumour diagnosis and ARAT treatment for mCRPC management. Some patients had missing data: nine for the radiological response (one in the AbA group and eight in the ENZ group), eight for the PSA response (concerning patients in the ENZ group), four for the extent of disease at PC diagnosis (one in the AbA group and three in the ENZ group) and one for the primary cancer therapy (one patient in the ENZ group). * Data presented as mean ± standard deviation. ** Data presented as median [range]. Abbreviations: AbA, abiraterone acetate; ARAT, androgen receptor axis-targeted; ECOG PS, Eastern Cooperative Oncology Group Performance Status; ENZ, enzalutamide; mCRPC, metastatic castration-resistant prostate cancer; PC, prostate cancer; PSA, prostate-specific antigen.

**Table 2:** Genotype distribution of the evaluated SNPs.

SNP	Genotype	AbA (N,%)	ENZ (N,%)	Minor/Major Allele Frequencies				
				AbA	ENZ	Total	CEU (European) *	IBS (Iberian) *
*CYP17A1* rs2486758	TT	25 (62.5)	59 (71.1)	0.21/0.79 (C/T)	0.17/0.83 (C/T)	0.18/0.82 (C/T)	0.23/0.77 (C/T)	0.18/0.82 (C/T)
	TC	13 (32.5)	20 (24.1)					
	CC	2(5.0)	4(4.8)					
*HSD3B1* rs1047303	AA	17 (42.5)	42 (50.6)	0.31/0.69 (C/A)	0.28/0.72 (C/A)	0.29/0.71 (C/A)	0.34/0.66 (C/A)	0.37/0.63 (C/A)
	AC	21 (52.5)	36 (43.4)					
	CC	2(5.0)	5(6.0)					
*YBX1* rs12030724	AA	33 (82.5)	71 (85.5)	0.09/0.91 (T/A)	0.07/0.93 (T/A)	0.08/0.92 (T/A)	0.12/0.88 (T/A)	0.13/0.87 (T/A)
	AT	7 (17.5)	12 (14.5)					
	TT	-	-					
*YBX1* rs10493112	AA	9 (22.5)	16 (19.3)	0.49/0.51 (C/A)	0.42/0.58 (A/C)	0.45/0.55 (A/C)	0.46/0.54 (A/C)	0.47/0.53 (C/A)
	AC	23 (57.6)	37 (44.6)					
	CC	8 (20.0)	30 (36.1)					

* Allele frequencies retrieved from Ensembl database (accessed on 8 January 2023). Abbreviations: AbA, abiraterone acetate; ENZ, enzalutamide; SNP, single-nucleotide polymorphism.

**Table 3:** Multivariate analysis for the risk of death among mCRPC patients with non-metastatic PC at cancer diagnosis and treated with AbA for mCRPC management (N = 24).

Variable	aHR	95% CI	*p*-Value
*HSD3B1 rs1047303* (CC/CA vs. AA ^1^)	3.28	**1.07–9.95**	**0.037**
Gleason score (>7 vs. ≤7 ^1^)	1.05	0.28–3.90	0.943
PSA levels at PC diagnosis (≥18 vs. <18 ng/mL ^1^)	1.35	0.39–4.73	0.637
*YBX1* rs12030724 (AT vs. AA ^1^)	4.43	**1.20–16.28**	**0.025**
Gleason score (>7 vs. ≤7 ^1^)	2.33	0.72–7.57	0.161
PSA levels at PC diagnosis (≥18 vs. <18 ng/mL ^1^)	0.81	0.26–2.58	0.723
*YBX1* rs10493112 (CC vs. AA/AC ^1^)	5.50	**1.27–23.79**	**0.022**
Gleason score (>7 vs. ≤7 ^1^)	1.29	0.37–4.51	0.693
PSA levels at PC diagnosis (≥18 vs. <18 ng/mL ^1^)	2.18	0.53–9.01	0.282

Bold values were considered statistically significant. ^1^ Reference group. Abbreviations: AbA, abiraterone acetate; aHR, adjusted hazard ratio; CI, confidence interval; mCRPC, metastatic castration-resistant prostate cancer; PC, prostate cancer; PSA, prostate serum antigen.

## Data Availability

The data presented in this study are available on request from the corresponding author.
